# Properties of Fibrous Concrete Made with Plastic Optical Fibers from E-Waste

**DOI:** 10.3390/ma13102414

**Published:** 2020-05-25

**Authors:** Zbigniew Suchorab, Małgorzata Franus, Danuta Barnat-Hunek

**Affiliations:** 1 Faculty of Environmental Engineering, Lublin University of Technology, ul. Nadbystrzycka 40B, 20-618 Lublin, Poland; 2 Faculty of Civil Engineering and Architecture, Lublin University of Technology, ul. Nadbystrzycka 40, 20-618 Lublin, Poland; m.franus@pollub.pl (M.F.); d.barnat-hunek@pollub.pl (D.B.-H.)

**Keywords:** concrete, fibers, porous materials, e-waste, utilization, physical parameters, mechanical parameters

## Abstract

This article presents research results relating to the potential for waste utilization in the form of polymer optical fiber (POF) scraps. This material is difficult to recycle due to its diverse construction. Three different volumes of POF were used in concrete in these tests: 1%, 2%, and 3%. The experimental studies investigated the basic properties of the concrete, the elastic and dynamic moduli, as well as deformation and deflection of reinforced beams. The microstructures, including the interfacial transition zones (ITZs), were recorded and analyzed using a scanning electron microscope. It was observed that 180 freezing–thawing cycles reduced the concrete frost resistance containing 3% POFs by half compared to the control concrete. The resistance to salt crystallization of this concrete decreased by about 55%. POFs have significant effects on the splitting tensile and flexural strengths compared to the compressive strength. The control beams were destroyed during the four-point static bending tests at half the force applied to the beams that were reinforced with POFs.

## 1. Introduction

Concrete is the most widely used construction material in the world. It is predicted that demand for concrete will increase by 12%–23% by 2050 compared to 2014 [1]. Despite its popularity, one must be aware that it exhibits poor tensile and flexural properties, which are desirable in the construction industry [2,3,4]. The requirements for structures in terms of the load-bearing capacity and serviceability are increasing under diverse static and dynamic conditions, and fulfillment of these requirements with ordinary concrete is becoming almost impossible. The brittleness of concrete is the cause of the formation and propagation of cracks in construction [5]. This necessitates the search for new types of concrete, the physical and mechanical properties of which must meet the current requirements. In addition, concrete production has a negative impact on the environment through greenhouse gas emissions, increased energy consumption, and overuse of the natural resources. Therefore, in order to promote sustainable concrete production, improvement of its properties by using innovative components should also be taken into account [6,7]. One of the ways to improve the properties of concrete and increase the bending strength of concrete structures is the use of various additives in the form of steel fibers [8,9], glass [10], fibers of natural and organic origin [11], as well as polymer fibers, i.e., polypropylene fibers [12,13] made of polyalcohol vinyl [14]. These fibers have become an effective material for strengthening cement materials, and for health-related and economic reasons are an alternative to asbestos, steel, and glass fibers [15]. They have many advantages, including strong chemical stability in alkaline and aggressive environments, light weight, convenient storage and handling, and electromagnetic transparency.

Fiber-reinforced concretes (FRC) have a number of advantages, including: greater compressive strength in the early stages of maturation than ordinary concrete [3]; greater tensile strength [16,17] higher bending and splitting tensile strength and ductility [18]; high dynamic resistance [19], reduction of crack propagation in structural elements [20,21]; better behavior during destruction, which does not occur rapidly [22]; and allowing the possibility of reducing traditional reinforcement [23]. Polypropylene-based fibers improve the fire resistance of concrete, which is often called the antispalling effect [24]. Concrete reinforced with polypropylene fibers is a functional, safe, and cost-effective design solution for roads, especially inside tunnels [25]. The presence of polypropylene-based draw-wired fibers in concrete significantly improves its resistance to cracks, while increasing its strength and durability [26].

These materials also have high abrasion resistance and durability, comparable to that of plain concrete. The disadvantages of fiber composites are the relatively high cost of the fibers [4], deterioration of the mixture’s workability with an increase in their content, and a lack of refined methods for dimensioning of structural elements made of such fiber-composite [27]. This is why other concrete additives are constantly being sought. It seems that optical fibers, being a type of optical waveguide and electromagnetic wave transmission medium [28], could be an interesting alternative.

Optical fibers (OF) are made of two basic coaxial layers, i.e., the core, which is a transmission medium in which light moves, and the surrounding jacket. Most often, the core is made of quartz glass or a plastic with similar parameters, while the coat is made of a polymer with a refractive index less than the core factor. The entire fiber is surrounded by an outer protective jacket [29]. Several polymers such as polymethylmethacrylate (PMMA), polycarbonates (PC), polystyrene (PS), cyclic olefin copolymer, and amorphous fluoropolymer are used for the preparation of mechanically improved OF [30,31,32,33,34]. These types of fibers are called polymer optical fibers (POFs) [35]. Generally, the OFs are used in active and passive forms in diverse branches of science and engineering [36]. The use of the OFs in biomedical technology can be categorized into three types: monitoring of physical parameters, imaging, and surgical operation [37]. They are especially used in medical research, particularly endoscopic research [38]. Such devices are also used as guidewires [39] and in ultrasound, X-ray, and magnetic resonance imaging machines [40]. They are great at transmitting light due to the use of the electroluminescent light technology to transmit data over medium distances. Moreover, OFs are suitable for use as amplifiers and optical switches, photonic sensors, photonic memory, single photon sources, in microcavity lasers, as well as in full-color display systems [41]. In construction engineering, OFs can also be applied to detect the shrinkage and cracking of concrete, as well as to monitor strains or temperature changes [42,43]. The use of the optical fibers ensures high throughput, ease of installation, and the replacement of copper cables with fiber optics. In comparison with traditional copper cables, the physical properties of POFs provide a number of advantages due to their light weight and flexibility. Additionally, they remain mechanically stretchable—in contrast to fiberglass—as well as allow easy and cheap connection with a large core [44]. Optical fibers are more commonly applied, and therefore increasing amounts of waste is produced in the form of useless cable scraps. For example, a single Japanese company recycles about 130 tons of waste optical cables annually [45]. European optic fiber waste production according to Zackrisson [46] in 2012 was evaluated at about 61,500 tons; thus, there is a need to devise recycling methods. Traditional cable recycling is focused mainly on metal recovery, while the polymer jacket is considered waste, stored, or incinerated. At present, increasingly restrictive regulations and concern for the natural environment mean that it is necessary to minimize waste disposal in landfills [47,48]. In the case of optical fibers, the problem of reprocessing is complex due to their composition. They contain fiberglass, polyethylene, copper wire, linear low-density polyethylene (LLDPE), polybutylene terephthalate (PBT), gels, ripcords, paper, and in the case of older cables, aluminum or steel. Therefore, cable optical fiber recovery and recycling is difficult due to technical and economic reasons. One method of recycling them is to break them down into components. This process involves the mechanical removal of the outer plastic protective layer, the separation of amide fibers, and the use of chemical solvents to remove the gel and recover the core, which is made of fiberglass or plastic. Unfortunately, this process is expensive, which means that the fiber waste is not recycled, but instead stored in landfills and incinerated, processes which also have associated costs. One of the best ways to manage solid plastic waste is recycling rather than incineration, which contributes to reducing waste and environmental emissions [49].

To the best of our knowledge, in the literature there are no examples of the use of POFs for dispersed reinforcement in concrete and reinforced concrete structures. Therefore, this study focused on the evaluation of the modification of concrete by POFs. The aim of this work was POF recycling through their usage as a supplementary material for concrete. This reduces the amount of waste stored and increases the tensile strength of concrete. The quality of the obtained concretes supplemented with plastic optical fibers was evaluated within the investigation of their physical and mechanical properties, such as their bulk density, open porosity, flexural tensile strength, compressive strength, splitting tensile strength, static and dynamic modulus of elasticity, frost and salt resistance, and other properties.

Thus, by considering the economic and ecological aspects of the decrease in e-waste, its application in the technology of concrete production is a reasonable choice for reinforcing concrete.

## 2. Materials and Methods

### 2.1. Material Properties

In order to determine the influence of plastic optical fibers (POF) (LSN, Lublin, Poland) from e-waste on the physical and mechanical properties of concrete, four mixtures of concrete were developed, the compositions of which are presented in Table 1. The developed samples were marked as follows: C0: standard concrete without waste; C1, C2, C3: concrete containing 1%, 2%, and 3% POFs, respectively. The optimal water/cement (w/c) ratio was 0.45.

**Figure 1:**
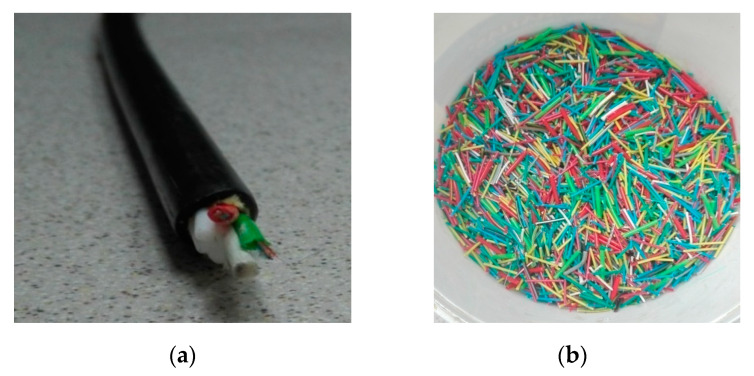
Optical fiber: (**a**) fiber optic cable; (**b**) POFs from e-waste, with lengths of 15–20 mm and diameter of 1 mm.

The following ingredients were used to prepare the mixture:

- The Portland cement CEM I 42.5R (Cemex Poland), tested according to PN-EN 196-1:2016-07 [50]: specific gravity of 3070 kg/m^3^, specific surface of 4049 cm^2^/g, compressive strength after 2 days of 31 MPa and after 28 days of 60.5 MPa, initial setting of 190 min, loss on ignition of 5.0% by weight of cement. The chemical ingredients of the cement are presented in Table 2.
- Plastic optical fibers (POFs) originated from the manufacturing plants located in Lublin, Poland (LSN). They consist of approximately 60% polyethylene and 40% copper cables. Thin fibers were obtained after removing the thick plastic cable jacket (Figure 1a). The POF was cut (15–20 mm, Figure 1b) and then analyzed in terms of certain physical properties, including: density = 1430 kg/m^3^, water absorption after 24 h = 0.01%, static modulus of elasticity = 10,000 MPa, tensile strength = 720 MPa.
- Coarse aggregate–river gravel with 2–8 mm and 8–16 mm granulation. The gravel aggregate was characterized by the following properties: apparent density = 2625 kg/m^3^, water absorption = 2.7%, resistance to fragmentation = 25, compressive strength = 190 MPa, elastic modulus = 6750 MPa, resistance to wear = 2.
- Fine aggregate–quartz sand with 0–2 mm granulation and specific density of 2650 kg/m^3^, bulk density of 1650 kg/m^3^, absorptivity of 0.4%, and mineral dust content of 0.4%.
- An efficient superplasticizer based on Glenium® SKY 591 polycarboxylate ethers was used at the amount of 0.70% in relation to the weight of cement in order to obtain the same workability in all the concrete mixtures.

### 2.2. Methods

The experimental methods used to determine various hardened concrete properties are presented in Table 3.

The concrete cube samples measuring 150 × 150 × 150 mm, 100 × 100 × 500 mm, and concrete cylinders (150 mm in diameter and 300 mm in height) were prepared according to EN 196-1:2016-07 [62]. The deviations of different specimens were ± 3 mm. All dry components except for polymer optical fibers were first mixed for 2 min; afterwards, POFs were added. Following another two minutes, water with superplasticizer was added and mixed for a subsequent 2 min. The samples were carefully observed to avoid fiber cluster formation in order to provide the best homogeneity of fiber allocation in the mixture. POFs were not dispersed into monofibers (elementary fibers) because thick optical fibers were enclosed in a polymer cable (Figure 1). A portion of each mixture was intended for use in consistency testing using the Ve-Be method [51]. This involves placing and compacting the concrete mixture in the form of a truncated cone. The mold is inserted into a cylinder 240 mm in diameter and 200 mm high attached to a vibrating table. A hopper is placed over the mold. The mold is filled with three layers, whereby each layer is compacted by hitting it with the striking bar 25 times. The bottom layer is compacted so that the engravings reach the base. After laying the third layer of the mixture, the hopper is removed. After compaction of the last layer, the excess concrete mix is removed with a sticking rod, so that the surface of the concrete mix is at the level of the upper edge of the mold. Demolding relies on uniformly lifting the mold up in about 5–10 s. After removing the mold, the transparent disc is lowered onto the upper surface of the concrete mix and the vibrating table is activated. The time is measured from the moment the vibrating table is turned on until the bottom surface of the disc contacts the concrete mix completely. The consistency class is determined based on the measured time in accordance with EN 12350-3 standard [51]. The density of all fresh mixtures was examined according to PN-EN 12350-6 standard [54]. The remainder of the mixtures was intended for molding. Steel molds were coated with a release agent, filled to half of their capacity, and compacted for 1 min on a flow table. Afterwards, a second layer of mixture was laid and the samples were compacted again. The samples were stored under laboratory conditions for 24 h and then removed from the mold and put into a water bath at 22.5 °C for 28 days. After maturing in water, the samples were pulled out and dried with a cloth. Six samples were used for each test in each series.

**Figure 2:**
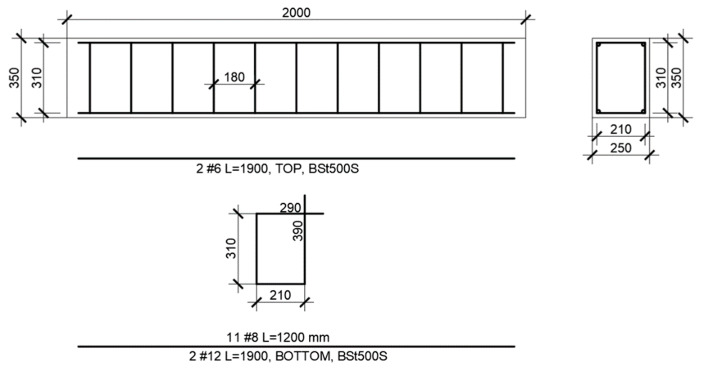
Reinforcement bars for the beams.

Four series of concrete samples (0, 1, 2 and 3) measuring150 × 150 × 150 mm (Figure 2) and 100 × 100 × 500 mm were prepared to determine the flexural tensile strength. 

### 2.3. Static and Dynamic Modulus of Elasticity

Testing of the static and dynamic modulus of elasticity was carried out on cylinders (150 mm in diameter and 300 mm in height). The dynamic modulus of elasticity of the tested samples was examined with the dynamic method utilizing the resonance frequency measurements generated by the C311-R frequency counter. Testing was conducted using the approximate output voltage at the level of 1 V.

The investigation involved installing an accelerometer on a silicone-coated concrete cylinder, which was then connected to the data acquisition system. During the test, a steel ball with a small diameter was employed as a source of impact. The steel ball was dropped onto the cylinder, while the vertical motion was measured using the accelerometer. The relevant data were acquired by means of computer software and the data acquisition system. This enabled the acquisition of amplitude–frequency and amplitude–time graphs. On the basis of the former graph, the peak value was obtained, indicating the resonant frequency of the composite specimen. The formula (1) below [48] was used to calculate the dynamic modulus of the considered concrete:

(1) $$E_{DM}=4L^{2}n^{2}\rho$$

where *E_DM_* is the dynamic modulus of elasticity, measured in GPa; *L* is the length of the specimen, measured in m; *n* is the frequency, measured in kHz; and *ρ* is the apparent density, measured in kg/m^3^.

### 2.4. Scanning Electron Microscopy Using Energy Dispersive Spectroscopy (SEM/EDS) Analysis

The morphology of the concrete with the addition of optical fibers was determined using scanning microscopy (SEM) (FEG Quanta 250 microscope, FEI, Hilsboro, OR, USA) equipped with a chemical composition analysis system based on EDAX X-ray energy dispersion. The samples for SEM testing in the form of a powder were glued onto the carbon holder using carbon glue (SPI Supplies, West Chester, PA, USA). Then, the preparations were sprayed with a carbon layer about 50 nm thick in a sprayer to achieve conductivity on the sample surface.

### 2.5. Ad Hoc Testing of Reinforced Concrete Beams at a Semitechnical Scale

The deformation and deflection examination of the concrete in the reinforced concrete model (with the dimensions of 2000 × 250 × 350) were conducted using each type of concrete (C0–C3). The beams were reinforced by 2 Ø 6 mm rods in the top and 2 Ø 12 mm rods in the bottom made of BSt500S steel, with a yield point Re = 500 MPa, while the stirrups were made of ribbed Ø 8 mm steel. The schematic view of the beam reinforcements is presented in Figure 2.

**Figure 3:**
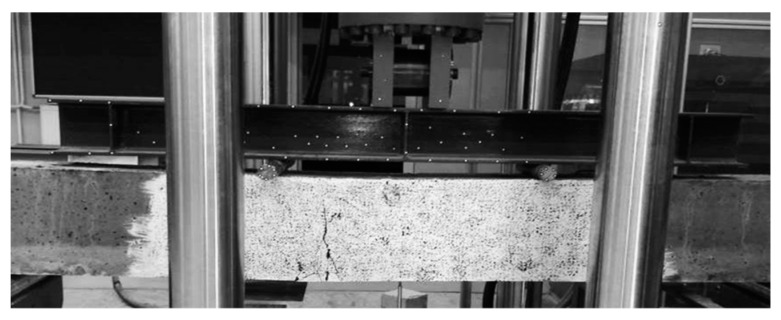
Experimental setup for the four-point static bending tests.

Four-point bending tests were performed after 28 days on a MTS 809 (MTS Systems Corporation, Eden Prairie, MN, USA) axial–torsional testing system machine, in accordance with ASTM C 78 [63]. The test involved four-point bending of beams through a rigid steel beam with supports in the form of steel rollers. The load consisted of two concentrated forces applied on one-third of the beam span. The static scheme was a simply supported beam. The method of loading the beam is shown in Figure 3. 

The control beams were made using the C0 concrete based on natural aggregate. Three beams were made for each concrete. The load was applied using a 2000 N capacity hydraulic jack at a rate of approximately 3 mm/min. Additionally, continuous measurement of displacement was carried out using the dial indicators and indirectly by means of highly accurate laser readings. The midspan deflection was measured using a laser optical displacement (LOD) device. The loads and strain readings were captured using the System 5000 data logger (Envco, Auckland, New Zealand). The deflections of beams were read at upper and lower levels, while their values at the upper, compressed, and lower tensile levels were read every 5 kN. 

## 3. Results

### 3.1. Physical and Mechanical Properties of Tested Concretes

**Figure 4:**
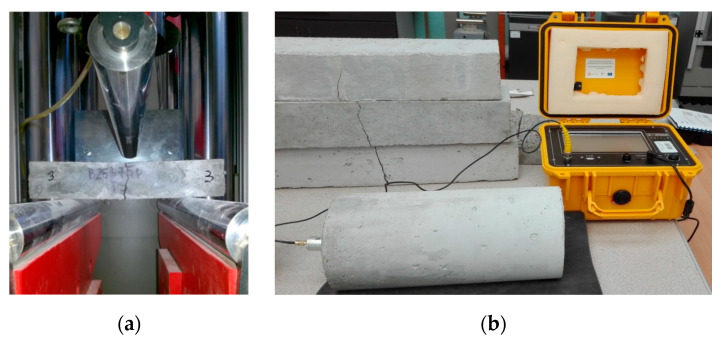
Samples during testing of the (**a**) flexural tensile strength; and (**b**) dynamic modulus of elasticity.

**Figure 5:**
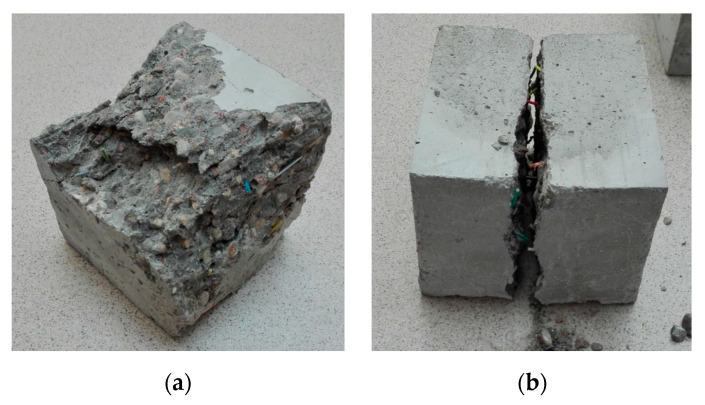
Cubic samples after testing of the (**a**) compressive strength and (**b**) splitting tensile strength.

Table 4 and Table 5 present the physical and mechanical properties of all analyzed concretes and the physical properties of fresh mixtures supplemented with POF, whereas Figure 4 and Figure 5 present the photographs of the tested samples during and after testing of the mechanical properties.

### 3.2. Results of the Ad Hoc Testing

**Figure 6:**
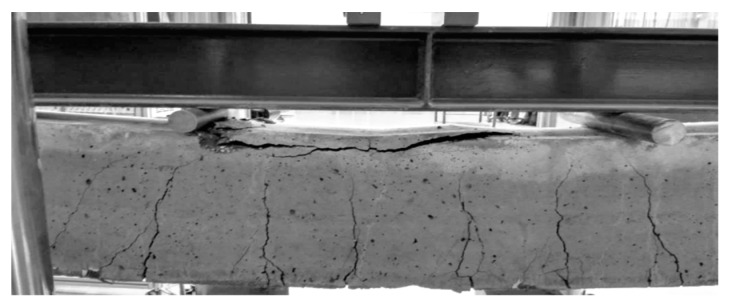
Image of reference beam damage in C1 concrete.

Figure 6 presents the damage in an exemplary beam after the four-point bending tests.

**Figure 7:**
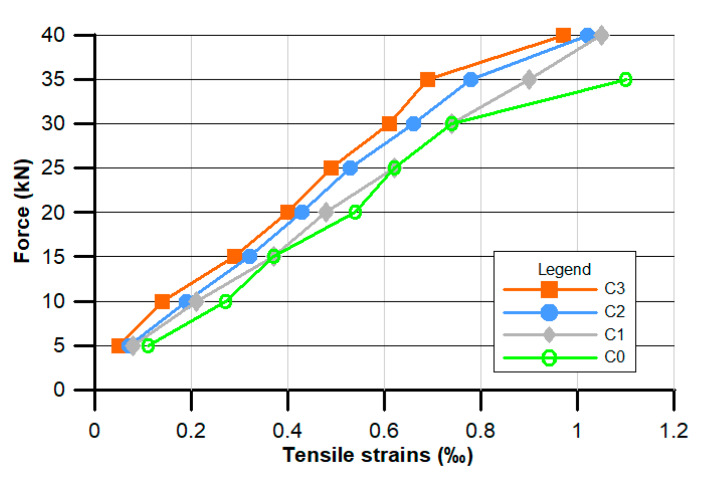
Experimental relationships between concentrated force F and tensile strain ε.

**Figure 8:**
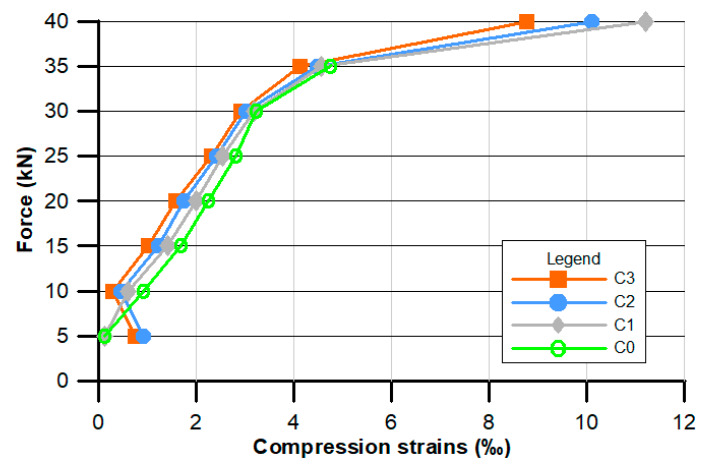
Experimental relationships between concentrated force F and compressive strain ε.

**Figure 9:**
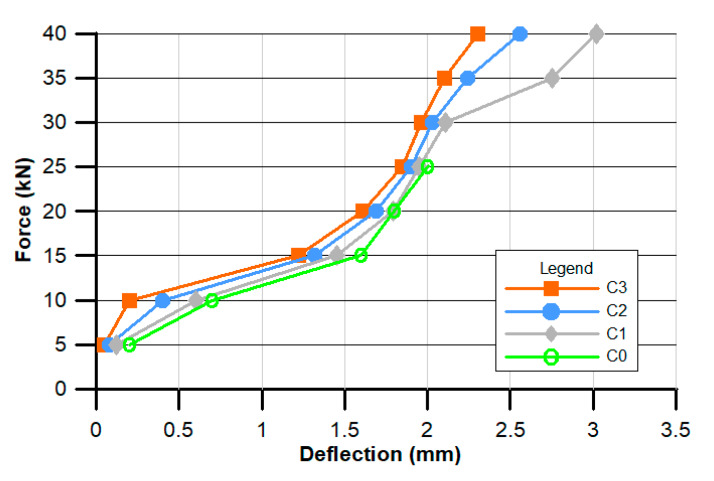
Experimental relationships between concentrated force and deflection.

Figure 7, Figure 8 and Figure 9 present the results of ad hoc testing of the examined beams. The measurements were conducted using displacement sensors and were performed after each 5 kN load increase. 

Figure 7 shows the mean values of three readouts of the tensile strain ε in relation to the applied force.

Figure 8 presents the mean values of the compression strain ε in relation to the applied force.

The force–deflection relationship is presented in Figure 9.

### 3.3. Microstructure of the Tested POF Concretes

**Figure 10:**
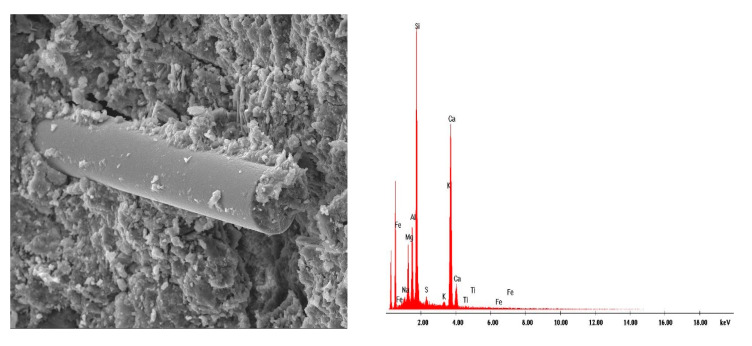
Single POF bundle structure in a cement matrix and elemental analysis results in the EDS micromatrix of a cement matrix at 100X magnification.

**Figure 11:**
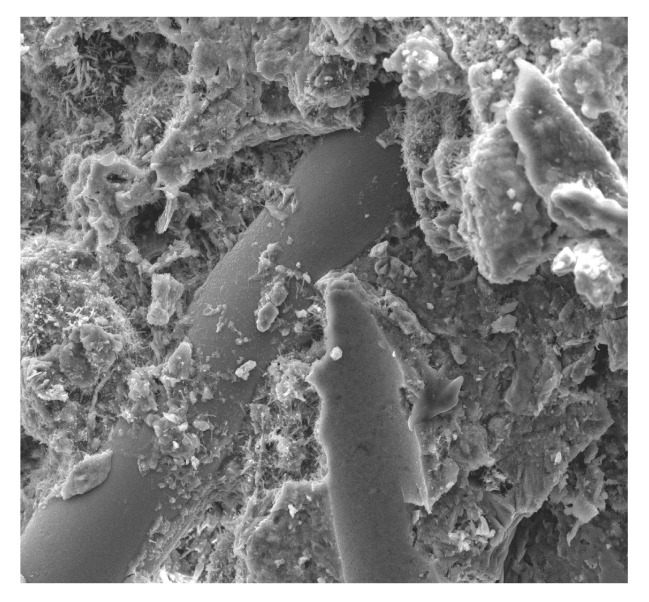
Fibers in the matrix surrounded by numerous pores at 100× magnification.

**Figure 12:**
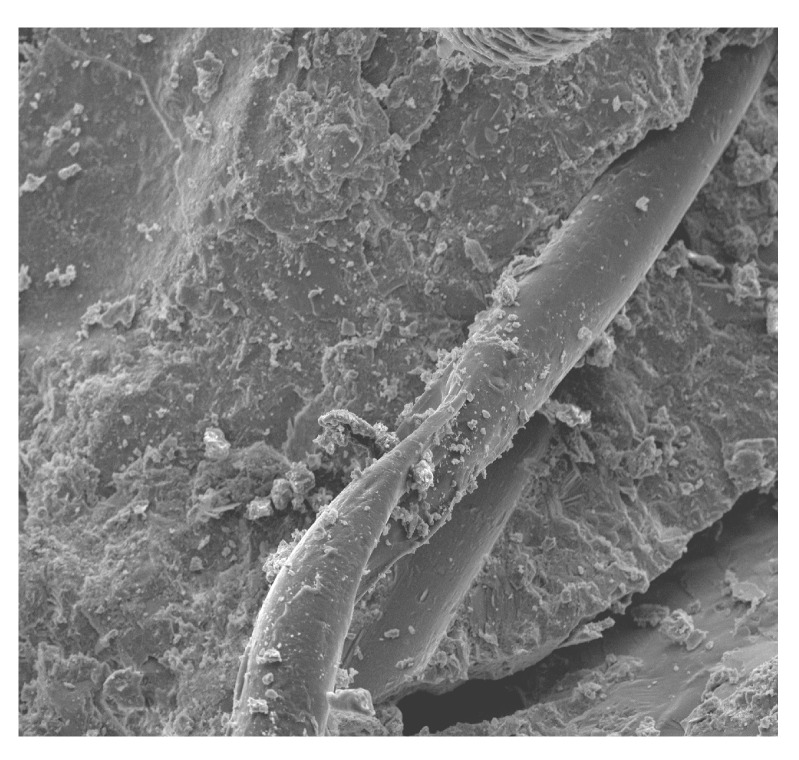
A fiber in the matrix and a scratch in the cement matrix at 100× magnification.

**Figure 13:**
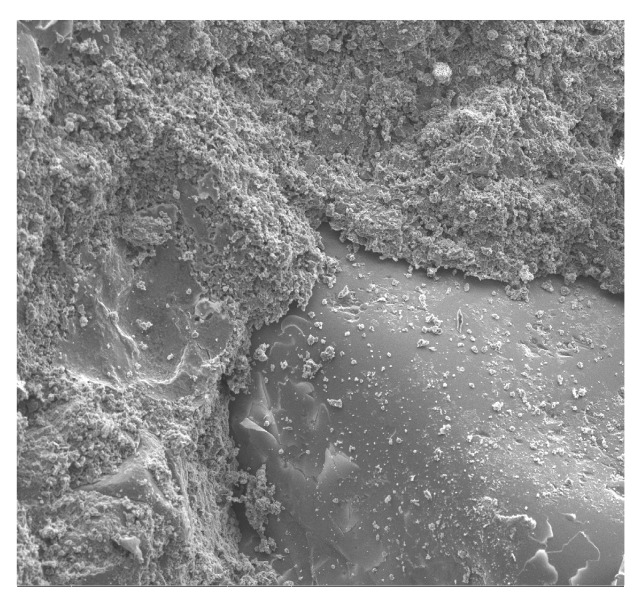
Contact zone of the cement paste with natural sand aggregate at 500× magnification.

**Figure 14:**
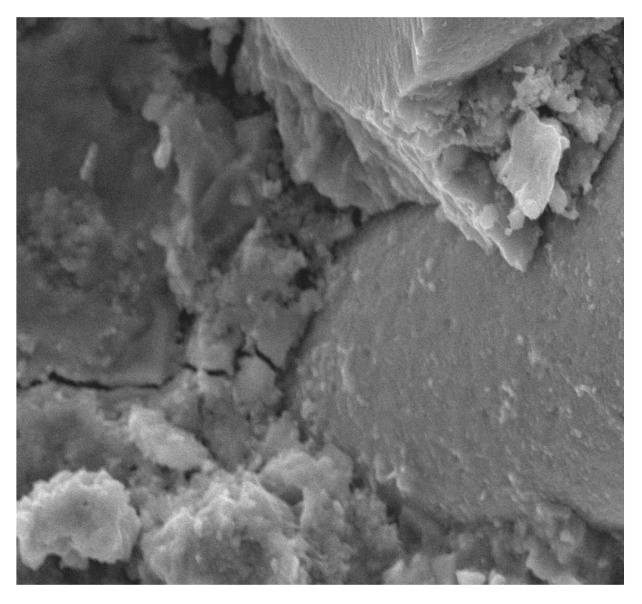
Microcracking near the optical fiber at 1000× magnification.

**Figure 15:**
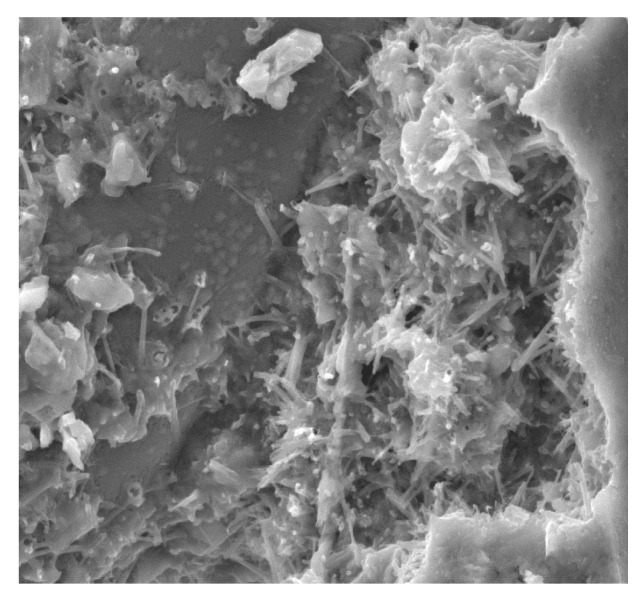
Ettringite crystals formed in close proximity to POFs at 1000× magnification.

**Figure 16:**
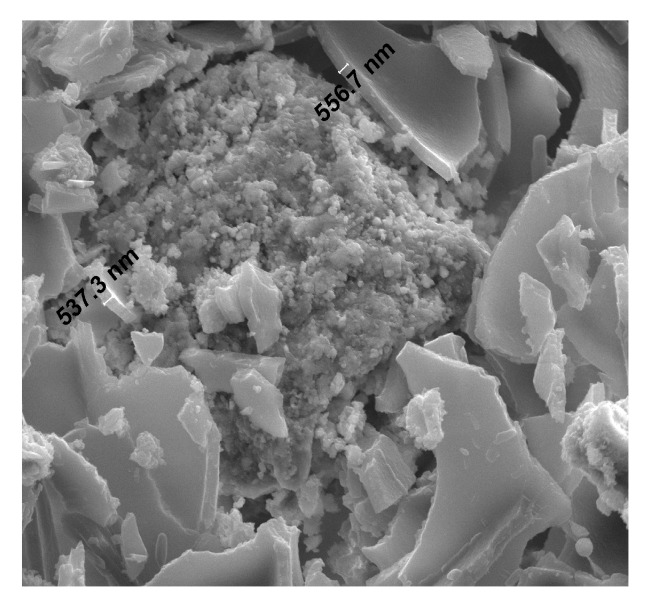
Compact calcium silicate hydrate (C-S-H) phase surrounded by the Ca(OH)_2_ portlandite crystals at 1000× magnification.

**Figure 17:**
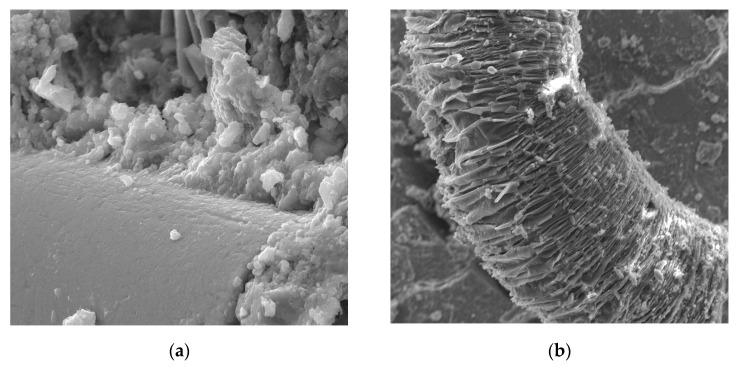
Contact zone of cement paste with POF after four-point static bending test: (**a**) 20,000× magnification; (**b**) 10,000× magnification.

Figure 10, Figure 11, Figure 12, Figure 13, Figure 14, Figure 15, Figure 16 and Figure 17 present the microstructure, interfacial transition zone (ITZ), and adhesion of cement paste to the POFs and aggregates. The image of the concrete microstructure is supplemented with the EDS graphs, presenting the chemical composition of the analyzed sample (Figure 10).

## 4. Discussion

### 4.1. Discussion on the Physical and Mechanical Properties of the Tested Concretes

From the data presented in Table 4, it can be observed that together with the increase of the amount of fibers in the concrete composition, the density of fresh mixtures and bulk density of the material decreases gradually, and in case of the mostly supplemented C3 sample, the bulk density decrease reaches about 11.5% compared to the C0 sample. Together with the decrease in bulk density, the open porosity and water absorption increase. The consistency time was extended from 21 to 31 s, along with an increase of the amount of POFs. In the case of the C0–C2 concretes, the same consistency class was achieved (V1). In the case of 3% POF additive, the consistency changed into V0, although the density decreased. The fibers caused the consistency change.

**Figure 18:**
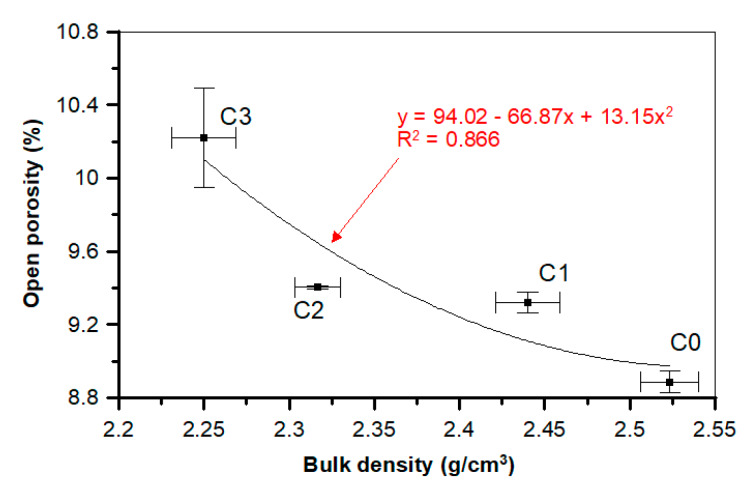
Relationship between the concrete’s bulk density and open porosity.

In the case of the mostly POF-supplemented C3 sample, the increase of open porosity equaled 14.8% and water absorption equaled 55.1% compared to the reference C0 sample. Figure 18 presents the dependence between bulk density and open porosity. The relationship between those two parameters can be described by the polynomial second-order model, with the determination coefficient being equal to 0.866.

The results for water absorptivity indicate the increased porosity of the mixtures due to the application of fibers, especially steel ones, which was demonstrated by other researchers [64] in studies on concretes supplemented with steel and basalt fibers. The authors of [64] showed that lower fiber-reinforced concrete strength should be combined with a two-fold increase in air content in this mix, as well as higher open porosity in the hardened concretes. Another study showed that highest porosity and absorptivity were observed in the cement mortars with the highest amounts of basalt fibers (1.5%), which were 29% and 56% higher than in standard mortar, respectively [65]. Quattrociocchi et al. [66] observed that the dispersion of basalt fibers proved to be more difficult, because the workability of the mortars decreased as the amount of basalt fibers increased (up to 6%) in the cement mix. It was necessary to add more water to the mix, thereby increasing the porosity. This reduction in workability was caused by a spatially crosslinked structure due to the random distribution of fibers in the mortar, which was also demonstrated by Ma et al. [67]. Glinicki [68] and Zhu et al. [69] recommended that the negative effect of fibers on the workability of the concrete mix could be compensated by an increased dose of the plasticizing admixture or by using a superplasticizer with a stronger liquefying effect instead of increasing the water content. 

The surfaces of POFs have hydrophobic properties, meaning they do not absorb water or interfere with the hydration reaction of concrete [59]. However, during drying, shrinkage occurs in hardened concrete, which causes cracks to develop over time, weakening the water resistance and exposing the concrete microstructure to destructive substances [70,71].

The mechanical data for particular concrete compositions are presented in Table 5. The results show a higher effectiveness of POF in boosting the concrete strength, with the exception of the compressive strength, which decreases. The flexural tensile strength increase in the case of the mostly supplemented sample equals 22.0% and the splitting tensile strength increase equals 16.9% compared to the C0 sample. Compressive strength decreases together with the amount of POF in the material, and in the case of the C3 sample the compressive strength reduction reaches 24.9% compared to the C0 recipe. The reduction in strength were minimized and the improvement of durability were achieved through the incorporation of nanomaterials in the concrete composition, which was proven in the research by Meng and Khayat [72,73], where graphite nanoplatelets (GNPs) and carbon nanofibers (CNFs) were used to increase the strength of ultra-high-performance concretes.

As shown in Table 5, the use of POF had a negative effect on the static and dynamic moduli of elasticity of the C1–C3 concretes. However, as expected, there was a correlation between the measured modulus of elasticity and compressive strength of a concrete. Smarzewski and Barnat-Hunek [74] observed a decrease in the compressive strength and modulus of elasticity in high-strength concretes with polypropylene fibers. The compressive strength of the reference concrete was 57.3% higher than the strength of the concrete with a 1% volume polypropylene fibers. The addition of polypropylene fibers at 1% volume caused an increase in the splitting tensile strength by 14% and a decrease in the static and dynamic moduli of elasticity by 10% compared to ultra-high-performance concrete without fibers [74].

Kim et al. [75] tested short polyethylene terephthalate (PET) fibers from recycled bottles added to concrete at 1% volume and found a decrease in the modulus of elasticity and compressive strength. However, the formation of shrinkage cracks during the maturation of concrete was significantly hampered by the PET fibers that bridged the cracks. Due to a decrease in compressive strength, Palou et al. recommended that the proportion of basalt fibers in the mortar ought to be in the range of 0.1% to 1.5% of the cement mass [76].

The most important goal of the optical fiber cable addition to concrete was to assess the potential of using the recycled fibers as dispersed reinforcement for concrete and to change the nature of the material operation at loading points. This effect is visible in the comparison of bending tests on the beams with the addition of the optical fiber cable and concrete without reinforcement.

Studies have shown a significant effect of fibers on the ductility of concrete by changing the nature of its performance from fragile to quasi-plastic. The fibers evenly dispersed in the concrete bridge its discontinuous structure, which results from the microcracks or scratches in the matrix. In the case of the non-reinforced concrete, a large concentration of stress is observed within the crack, which negatively affects the strength of this zone. The presence of the fibers allows the transmission of forces resulting from loads from one side of the crack to the other, which significantly reduces the stress concentration on the edge of the scratch. This phenomenon limits the transformation of microscratches into larger cracks, preserving the integrity of the concrete element with POFs after scratching. Unlike ordinary concrete, POF concrete does not behave in the same way as a brittle material, but exhibits quasi-plastic properties. Therefore, it seems that optical fiber cables are most effective after the brittle matrix breaks, similarly to steel fibers. According to Naaman and Reinhardt [77], the observed flexural behavior can be attributed to the fiber length and bond strength. This bridging stress may arrest the further propagation of the macrocracks. As a result, the toughness of the material may increase.

The use of steel fibers increases both the compressive and flexural strength, as demonstrated by many authors [70,74,75]. Using the steel fibers (dosage 25 kg/m^3^), Grzymski and Musiał [78] observed an increase in tensile strength for the beams by about 30%, which is in line with expectations. Steel and other fibers bridge cracks due to the lateral expansion of the concrete during bending. As the crack width increases, fibers are pulled out of the cement matrix, which results in increased material strength [78,79,80].

As can be seen in the research of Kalpana and Tayu [4], who studied the lightweight perlite concrete supplemented with industrial steel waste, the increase in the steel waste content to 0.5%, improved the compressive strength by 13% compared to concretes without fibers. Conversely, adding more steel waste (1%) deteriorated the compressive strength by 7%, below the strength of the reference concrete. This decrease in strength at higher fiber contents may be due to the problem of evenly mixing the steel waste in the lightweight perlite concrete, because the shape and size of steel waste is irregular [79]. Semenov et al. [81] reported that in the case of 1% addition of basalt fibers, the flexural strength increased by 55%, while Barnat-Hunek et al. reported a flexural strength increase of 30% with 1.5% addition of basalt fibers [65]. The use of steel waste in concrete increased the flexural strength—at 0.5% and 1% volume of steel waste, the flexural strength increased by an average of 23% and 52%, respectively, which was more than the control lightweight perlite concrete [79]. After scratching, the optical fiber elements were still capable of carrying loads and deforming, but loosening and disturbing the matrix structure led to a decrease in the compressive strength as the fiber content increased. During the compression test of the concrete samples, a brittle crack was observed passing through the entire middle section of the sample. The elements made using the recycled fibers could easily be separated into parts without the use of large forces or tools, because the fibers could easily be removed from the damaged matrix. A series of concrete samples without fibers held together despite having many cracks. The examples of destruction of the compressed elements are shown in Figure 11.

In the case of both static and dynamic moduli of elasticity, the addition of POF decreases their value. For the static modulus of elasticity, the decrease equals 15.6%, while for the dynamic modulus this amounts to 15.2% compared to the reference C0 sample.

**Figure 19:**
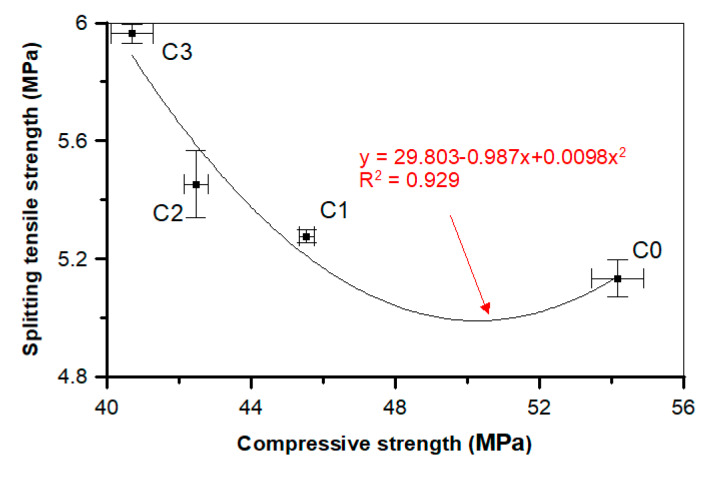
Relationship between the compressive and splitting tensile strengths of a concrete.

On the basis of the test results, the dependencies and associations between the different concretes with and without POF properties were proposed. The graph in Figure 19 shows the dependence between the compressive and splitting tensile strengths for the tested concretes. Research has shown that increasing the content of POFs in concrete decreases the compressive strength, but the fibers bridge the cracks formed during the splitting tensile strength and flexural tensile strength tests, which is also visible in Figure 5b. The fibers delay the appearance of the microcracks and bridge the macrocracks. The compressive strength corresponds to the splitting tensile strength of concrete with or without POF. The polynomial trend was characterized by a good determination coefficient (*R*^2^ = 0.929) and relatively low errors in the intercept. It is essential to point out that the amount of fibers had an influence on the data.

**Figure 20:**
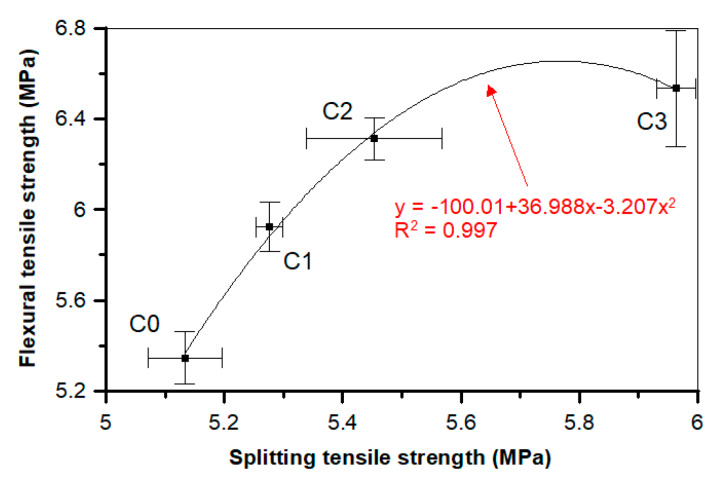
Relationship between the splitting tensile and flexural tensile strengths of a concrete.

The relationship between the flexural tensile and splitting tensile strengths for a concrete is presented in Figure 20. The relationship between the flexural tensile and splitting tensile strengths for a concrete with and without POF is presented in the form of the polynomial formula y = −100.01 + 36.988x − 3.207x^2^. The high determination coefficient equal to 0.997 indicates that the data have been matched by the best *R*^2^ value. A high determination coefficient value indicates that the flexural tensile strength has a strong association with the splitting tensile strength. There is a clear grouping of the results depending on the quantity of fibers in the concrete, which was also shown in the relationships between the compressive, splitting tensile, and flexural tensile strengths (Figure 19 and Figure 20).

**Figure 21:**
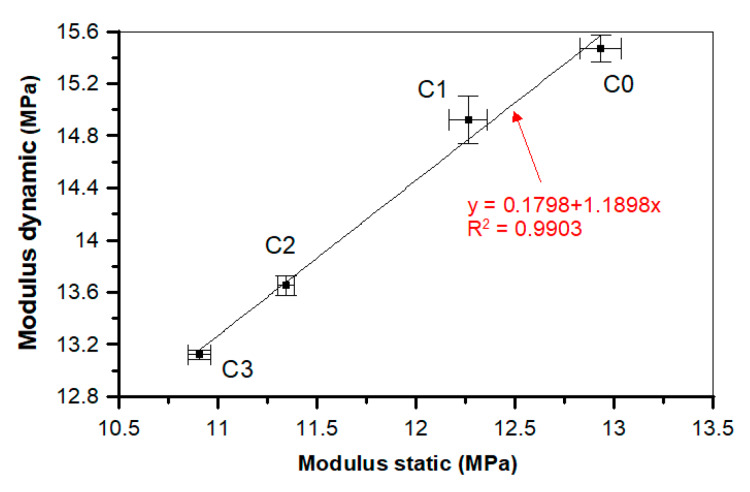
Correlation between the static and dynamic moduli of a concrete.

Figure 21 illustrates the dependence of the static and dynamic moduli of elasticity for all analyzed concretes. It was observed that the static modulus is completely congruous with the dynamic modulus. The linear trend was characterized by a good determination coefficient (*R*^2^ = 0.990) and low errors in the intercept.

**Figure 22:**
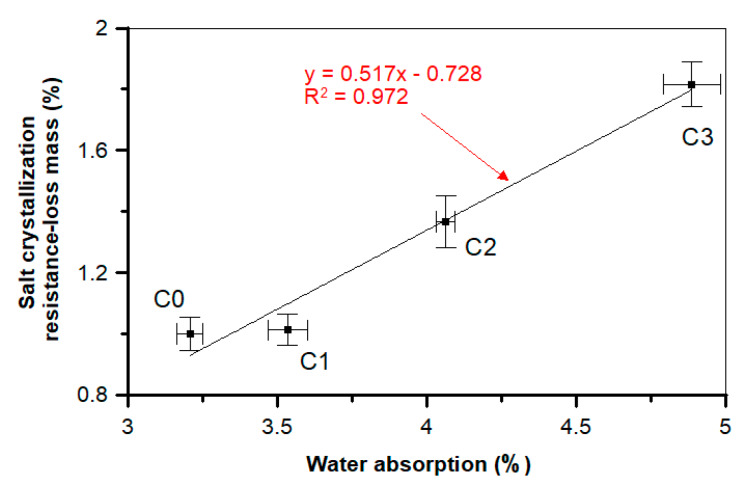
Relationship between the water absorption and salt crystallization resistance of a concrete.

An interesting relationship can be observed for the changes in water absorption of a material and mass loss after the salt crystallization test. The diagram presented in Figure 22 shows the linear dependence between those two factors, with a slope value equal to 0.517 and y-intercept value close to 0, which confirms a good linear dependence between those two values, additionally confirmed by a high determination coefficient (*R*^2^ = 0.973). During the salt crystallization resistance test, the mass loss reached 79.0% in the case of the C3 sample as compared to C0. In turn, no deterioration was observed in the ultra-high-performance concrete with steel and polypropylene fibers after the salt crystallization test [74].

The frost and salt resistance values are considered to be the main elements in the assessment of the concrete durability. When the pores in the concrete are almost completely saturated, freezing can cause severe cracks and damage to the concrete [64,65]. In the mortar studies with 1.5% basalt fibers, the mortar weight loss was three times greater, while the reduction in compressive strength was reduced by almost a factor of five [65]. Fenu et al. [82] and Jiang et al. [83] showed that the cement mortars with basalt fibers are characterized by lower workability, higher bending strength, and water permeability, but also by lower frost resistance due to higher porosity, which was also confirmed by the investigations performed in this study. In the case of the concrete reinforced with steel fibers, significant concrete damage occurred during cyclic freezing and thawing (F-T) [82]. In the concrete with steel fibers, the number and volume of the pores and voids between the fibers and the cement matrix increased. The main reason for the damage was expansive internal pressure during water freezing. Other studies showed that steel fibers do not slow down the propagation of the microcracks, meaning they do not protect against damage during freezing and thawing cycles [73]. In this experiment, the authors observed cracks on the sample surfaces and the corrosion of steel fibers. With the increase of the steel fiber content in the concrete (up to 1%), more cracks and voids were observed. The weight loss of this concrete was 22 times greater than the reference one. In the case of the polypropylene fibers [73], it was noted that the weight loss was 11 times greater than the reference concrete.

**Figure 23:**
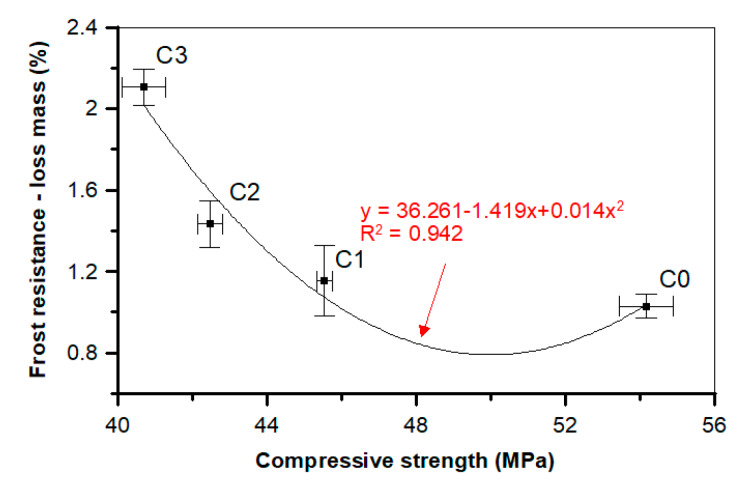
Relationship between compressive strength and frost resistance after 180 concrete freezing and thawing (F-T) cycles.

In our research the frost resistance features decrease (loss mass increases) together with POF supplementation (Table 5, Figure 23). In the case of the mostly supplemented composition, the mass loss after the test was the greatest; compared to the reference C0 sample, it was about 104.9% higher Additionally, a close dependence was observed between the compressive strength and mass loss after the F-T test (Figure 23). 

This can be described by the second-order polynomial model, which can be presented in the form of y = 36.261 − 1.419x + 0.014x^2^ formula. The high determination coefficient *R*^2^ equal to 0.942 indicates that the data were properly fitted. A higher correlation coefficient value indicates that the compressive strength has a strong relationship with frost durability. Together with the decrease of the compressive strength, the mass loss increases after the F-T test, which can be confirmed by the research of other scientists [64,65,84].

### 4.2. Discussion on the Ad Hoc Test Results

The main purpose of the fiber addition to the concrete is to change the nature of the material performance at a load causing tensile stress. This effect on the reinforced concrete and concrete beams without reinforcement is visible in the comparison of bending tests. When the tensile strength is achieved, a crack appears in the concrete beam that causes the destruction of the sample. After the cracking, the fiber-reinforced concrete beam goes into extra-elastic operation phase due to the cooperation of the fibers, allowing for further load transfer [78].

The POF supplementation influenced the value of the tensile strains in the tested beams made of POF concretes. As shown in Figure 7, in the case of the beam made from the C0 composition, which did not contain POFs, the loading force equal to 35 kN caused structural damage and caused the highest measured tensile strain value of approximately 1.1%. For the POF-supplemented concretes (C1, C2, and C3), the tensile strains were lower and equal to 0.9, 0.78, and 0.69 mm, respectively. For the loading force at the level of 40 kN the POF beams were still not destroyed, and in each case the tensile strains were close to 1 mm, which was still lower than that achieved by the C0 beam (which was not supplemented with POF).

In the case of compressive strain, the values of the beams manufactured from the supplemented concrete (C1–C3) were comparable to those of the traditional concrete (C0). For the smallest loading force (5 kN), the standard beam compression strain is the lowest. Together with the loading force increase, all strains are higher and the C0 concrete beam reaches slightly higher strain values. Interestingly, after reaching the value of 35 kN of loading force, the C0 beam was damaged, while C1–C3 beams still performed, reaching significantly higher compression strain values equal to 11.2% (C1), 10.1% (C2), and finally 8.78% (C3).

Finally, based on the test results shown in Figure 9, it can be stated that the deflection values for the POF concrete beams were higher than these for the standard concrete (C0) beams. In the case of the standard beam, the loading force of 25 kN caused a deflection value equal to 2 mm; the beam was damaged after application of a higher loading force. The beams made from the concrete supplemented with POF gave lower deflection values, with the C3 concrete giving a beam deflection value of about 1.9 mm. Higher loading forces did not cause the destruction of POF beams, they only induced higher deflection values. The beam with the least POF supplementation (C1) reached a maximum deflection value equal to 3.02 mm, while the most heavily supplemented beam (C3) only reached 2.3 mm.

According to Pająk and Ponikowski [85], the addition of the fibers affects the flexural tensile strain. The fracture energy increases along with the fiber addition. Fibers applied at 0.5%, 1.0%, and 1.5% of the volume ratio indicate that the largest deflection at the maximum load (equal to 0.92 mm) was recorded for the samples with 1.0% fiber addition, while for the 1.5% volume ratio the deflection at the maximum load was only 0.64 mm. Glinicki’s observations [68] of fracture toughness when bending fiberglass with synthetic microfibers showed that even with a significant deflection of the beams, the samples retained integrity over 1/100 of the span. After their forced tearing, it was found that the total destruction was associated with the extraction of synthetic fibers from the cement paste grout, while no fiber tearing was observed [68].

The analyses carried out by Stachniewicz et al. [86] during a bending test on concrete beams with the addition of steel fibers showed that the formation of the first crack did not lead to the sudden destruction of the element. Cracks in concrete elements supplemented with fibers appear at a higher load compared to concrete without fibers. As the critical crack develops, the tensile stress is absorbed by the fibers. Subsequent cracks in concrete lead to the further deformation of the element, but not to complete destruction [85]. The beneficial effects of various types of fibers on concrete properties are presented in previous articles [74,86,87,88,89]. Chunxiang and Patnaikuni [90] and Dihn et al. [91] recognized that at the ultimate limit state, the addition of steel fibers could partially or completely replace the traditional tensile or shear reinforcement. Fraternali et al. [92] showed that PET fibers from bottles in a concrete at 1% fiber volume caused a significant increase in the strength and ductility of fiber-reinforced concrete. Sadaqat and Tehmina [87] showed that self-compacting reinforced concrete beams supplemented with polyethylene terephthalate fibers and strips resisted greater loads with higher ductility. The load capacity was 13% higher than the reference beam, while the maximum load capacity was achieved with a smaller deflection. The fiber-reinforced concrete beams showed slight deflection before damage. The short length of the polyethylene terephthalate fibers helped control the cracks, and in this way the beams showed large deflections and more cracks before damage [86]. The beam containing PET strips cracked at a slightly lower loading force, but it was able to resist the additional load even after the first crack. The authors suggest that the PET strips may serve as a steel stirrup in concrete beams, which arrest the crack in the shear zone, meaning cracks will disseminate in the pure flexure zone. The strains in the tension zone of the reinforced beams were higher than the yield strain of the steel. The beam continued to take the load after cracking. This was because the PET fiber acted as reinforcement. The PET strips provided flexural resistance, with better ductility.

In general, the fibers have little effect on the compressive strength value under static conditions. A clear effect of the fibers on the post-peak parameters of the concrete was noticed. The fibers delay the appearance of the microcracks and fill the macrocracks. Under static conditions, the microcracks slowly spread into macrocracks [93]. However, in the case of dynamic loading, the concrete cracking time is very short. Immediately, microcracks are transformed into macrocracks and other microcracks appear. As a result, more energy is consumed and a higher value of compressive strength is observed [94,95,96]. Regarding the fibers, more of these participate under dynamic conditions than under static conditions. Thus, their operation is more pronounced in the case of dynamic load than under static load. As a result, the sensitivity to reinforced concrete strain with fibers is lower than the usual matrix under compression [97,98], which was also shown in our study.

### 4.3. Discussion on the Microstructure of the Tested POF Concretes

Most of a concrete’s properties, such as strength, liquid and gas permeability, or frost resistance, depend on its structure. These features result directly from its composition and the arrangement of the elements in the structure of the material. Therefore, the areas between POFs, aggregates, and grout grains and phases occurring in concrete were investigated.

The concrete morphology and elemental composition analysis performed with a scanning electron microscope and the EDS analysis indicate mainly calcium, aluminum, silicon, and iron oxides are present in the concrete. Due to its dominant share, the C–S–H phase, which is a component of cement slurry hydration products, constitutes an important component of the cement slurry (Figure 10). The conducted research shows that the hydration products in a concrete are mainly composed of irregular and isometric or flattened particles, forming compact clusters that correspond to the morphological types of C–S–H phases III and IV [99]. Flocculent C–S–H gels overlap, thereby increasing the mortar matrix density, strengthening the bond between the optical fiber and mortar matrix and improving the performance of the interfacial transition zone (ITZ). The compressive strength of the C–S–H gel is higher than that of the Ca(OH)_2_ crystals [100], which has been observed in a concrete (Figure 16). The analysis of the fracture photographs of the concrete samples indicates the presence of slender optical fiber surfaces with low roughness embedded in a cement matrix. It can be observed that the dispersed reinforcement in the form of optical fibers is not tightly wrapped by the cement slurry and that there are also small microcracks visible in the mortar matrix (Figure 10).

Similar observations were made by Barnat-Hunek et al. [65] in mortars made of basalt fibers, where higher mortar porosity and 1.5-µm-wide cracks appeared between the basalt fibers and cement paste. The slack contact zone between the fibers and cement paste caused a reduction in the compression strength, which was described in both the cited [65] and current study. POF is a polymer material. When POF is added to the cement mortar, it results in the formation of a liquid film at the interface of the POF and the cement paste. In view of the above information, one can conclude that the bond strength between the optical fiber surface and cement paste is poor, as shown in Figure 10, Figure 11 and Figure 12. A similar phenomenon was demonstrated in ultra-high-performance concrete with polypropylene fibers [84]. Additionally, the numerous pores between the polypropylene fibers and mortar lead to a notable increase in absorptivity, which was confirmed by the research presented in [63]. This is probably due to the presence of the ITZ zone between the cement paste and POFs, which is commonly found in the immediate vicinity of the aggregate grain surface. Due to the increased porosity caused by the so-called “wall effect”, the transition zone is generally considered to be the weakest element of the concrete microstructure, as demonstrated in the studies by Glinicki and Litorowicz [101]; by Golewski [102], who studied concrete containing fly ash; and by Barnat-Hunek et al., who investigated concrete containing nanocellulose [103]. The ITZ accumulates more mixing water due to cement grains being less tightly packed in this zone than at other distances from the surface. The fiber in the matrix is surrounded by numerous pores caused by air bubbles. In the ITZ zone, there are several voids between the mortar and the fiber (Figure 11), as demonstrated in [74]. The C-S-H gel particles on the fiber surfaces also help to enhance the bonding performance of the ITZ between the mortar and fibers (Figure 11). Similar observations were made by Barnat-Hunek et al. in high-strength mortars containing basalt fibers [65] and in self-compacting concrete [82].

There are slight microcracks in the cement matrix due to the excellent perforation of the ITZ bond between the mortar. However, a crack with a width of about 25 µm that does not occur at the contact of the fiber with the matrix but in the matrix itself can be seen at a distance of about 100 µm. Its appearance may be due to the fibers being pulled out of the concrete matrix during the destruction and not due to the fibers breaking or to the matrix shrinking during maturation (Figure 12). A similar phenomenon was observed by other authors [65]. It is generally accepted that the gap is formed by the plastic settlement, shrinkage of the concrete, or bleeding. Réunion Internationale des Laboratoires et Experts des Matériaux Technical Committee (RILEM TC 262-SCI) uses the term “settlement and bleeding zone” to describe macroscopic voids. Depending on the formation cause, they can be filled with air or liquid [104].

Ettringite crystals were observed near POF and between sulfate ions, which are formed during the reaction between hydrated calcium aluminates and calcium hydroxide and are a product of cement hydration. The crystals are over 5 µm long, which overgrow and intertwine to form the clusters (Figure 15). The presence of an increased amount of ettringite in the structure of the concrete transition zone may affect the weakening of the grout adhesion to POF. The resulting microscratches in the cement matrix near the optical fibers are caused by the ettringite swelling and by the porous structure of the ettringite itself (Figure 14). As shown by Lo and Cui [105], the pores in the structure of the ettringite form a network, with their size ranging from 0.3 to 1 µm, which is larger than the border pore size of 50 nm indicated by Mehta [106] as being important in terms of permeability and strength. With the addition of the optical fibers, the amount of free shrinkage can be reduced, which at the same time helps to avoid the formation of scratches in the concrete at a later stage, because a single crack in the concrete is replaced by a system of dispersed microscratches that do not have a negative effect on the strength of the material.

Figure 17 represents the POF status after the four-point static bending test. It can be observed that the POFs are in very good condition, as they were not damaged during the bending test. Figure 17a shows the ITZ zone, where no microcracks or cracks are visible and the slurry adheres very well to the fibers. In contrast, Figure 17b shows a rod that was significantly bent during the bending test. The rod was not damaged or broken and it bridged the cracks very well.

The observations made using the scanning microscope indicate the compact and solid structure of the grout and its very good adhesion to the fine aggregate–sand mixture (Figure 13). The paste morphologies near the aggregate grain and also at a considerable distance from it are similar. In many of the analyzed areas, the morphology is homogeneous. In the transition zone containing quartz grains in the natural sand, portlandite was not found. Randomly arranged portlandite plate crystals measuring about 20 µm long and 2 µm thick surround the C–S–H phase in Figure 16. There are no visible voids in the contact zone. This area does not show macroporosity, rather it is tightly filled near the separation boundaries. No scratches or gaps are observed, so there is good adhesion of the grout to the aggregate grains, which in turn do not reduce the strength parameters of the concrete. The better physical and chemical adhesion of the grout to the aggregate guarantees the possibility of cooperation of both components in transferring the stresses caused by all kinds of interactions and loads, which was confirmed in another work on the properties of high-performance concrete made with coal cinder and waste foundry sand [107]. As a result, it is possible to improve the mechanical and physical properties of concretes related to their tightness, which are not only caused by a concrete having a smaller number of pores, but also by microcracks in the cement matrix. This study indicates that the addition of POFs caused changes in the structure of the concrete, affecting the physical and mechanical parameters (e.g., causing higher porosity, lower compressive strength and moduli of elasticity; Table 4 and Table 5). Other studies using SEM results illustrated that adding a certain amount of steel and polypropylene fibers to concrete [74] and basalt fibers [65] to cement mortar considerably changed the microstructures. It was observed that the smallest microcracks in the ITZ between the cement paste and aggregate occurred in the concrete containing steel fibers, while larger cracks occurred in the concrete containing slippery polymer fibers.

## 5. Conclusions

The industrial POFs used in this study could be an economical and environmentally friendly solution. This study was performed to determine the effects of the addition of POFs on the physical and mechanical properties, as well as on the freezing–thawing and salt crystallization resistance of concrete. 

Through the analysis of the examination results, the following conclusions can be drawn:

- POFs cause higher concrete porosity, lowering compressive strength, as well as the static and dynamic moduli, in turn lowering the durability. The SEM studies showed that this is connected with the ITZ structure, located between the POF and the matrix.
- The increased volume of free pores in the concrete reinforced with POFs decreased the mass after the frost and salt crystallization test. It was observed that 180 freezing–thawing cycles caused a doubling of the concrete frost resistance with the addition of 3% volume POFs compared to the control concrete. The resistance to salt crystallization of C3 decreased by 55%. This was due to the increased porosity and absorptivity of concretes reinforced with POFs.
- The deflection values for the beams with 3% POFs were 25% lower than in the beams with 1% POFs. The control beams were destroyed at half the force (20 kN) compared to the beams with POFs.
- Microstructure studies illustrated that adding optical fibers to concrete considerably changes the microstructure. The presence of an increased amount of ettringite in the ITZ structure of the fiber-reinforced concrete can weaken the adhesion of the grout to the POFs; the microcracks are visible in this zone together with a larger number of pores, which decrease some of the strength parameters.
- POFs have a significant effect on the splitting tensile and flexural strengths as compared to the compressive strength. Thus, considering economic and ecological aspects of decreasing e-waste, its application in concrete production to reinforce concrete is a reasonable choice.
- Practical limitations in the application of concrete reinforced with POFs result from the fact that the frost resistance decreases with increasing POF content. Therefore, elements made of POF concrete should be used indoors. The second limitation is the decreased compressive strength, which to some extent is compensated for by the increase in splitting tensile and flexural strengths. Therefore, this concrete should rather be used in elements exposed to bending and tensile forces, rather than in elements exposed to compressive forces, such as columns or walls.

## Figures and Tables

**Table 1:** Composition of concrete with polymer optical fibers (POFs) (kg/m^3^).

Component	Unit	C0	C1	C2	C3
Portland cement CEM I 42.5 R	kg/m^3^	365	365	365	365
Coarse aggregate 2–8 mm	kg/m^3^	480	480	480	480
Coarse aggregate 8–16 mm	kg/m^3^	704	704	704	704
Fine aggregate/sand 0–2 mm	kg/m^3^	710	710	710	710
POF 15–20 mm	% m/v	0	1	2	3
	kg/m^3^	0	14.3	28.6	42.9
Superplasticizer	L/m^3^	2.55	2.55	2.55	2.55
Water	L/m^3^	165	165	165	165

**Table 2:** Chemical composition of cement CEM I 42.5R (%).

Ingredient	CaO	SiO_2_	Al_2_O_3_	Fe_2_O_3_	MgO	Na_2_O	K_2_O	Na_2_O_eq_
Content	65.61	20.73	4.41	3.34	2.40	0.33	0.51	0.51

**Table 3:** Experimental methods and properties of concrete with and without POFs.

Properties	Method	Properties	Method
Consistency of fresh mixture	EN 12350-3 [51]	Dynamic modulus of elasticity	ASTM C666 [52] ASTM C215-85 [53]
Density of mixtures	PN-EN 12350-6 [54]	Compressive strength	EN 12390-3 [55]
Bulk density	EN 12390-7:2019 [56]	Flexural tensile strength	EN 12390-5 [57]
Open porosity	PN-B-06250 [58]	Frost resistance	PN-B-06250 [58]
Water absorptivity	PN-B-06250 [58]	Splitting tensile strength	EN 12390-6:2001 [59]
Salt crystallization resistance	BS EN 12370:1999 [60] ASTM C215. A	Static modulus of elasticity	ASTM C469-02 02:2004 [61]

**Table 4:** Physical properties of the concretes.

Parameter	C0	C1	C2	C3
Density of mixture (kg/m^3^)	2590	2560	2450	2342
CV	3.4	2.6	3.7	4.1
Consistency time (s)	21	24	28	31
CV	1.0	0.3	1.0	0.5
Class	V1	V1	V1	V0
Bulk density *ρ* (kg/m^3^)	2536	2450	2319	2240
CV	2.3	2.3	2.1	2.2
Open porosity (%)	8.89	9.35	9.40	10.21
CV	0.5	0.5	0.6	0.7
Water absorption (%)	3.21	3.56	4.06	4.98
CV	0.60	0.54	0.23	0.31

CV = Coefficient of variation.

**Table 5:** Mechanical properties of the concretes.

Parameter	C0	C1	C2	C3
Compressive strength (MPa)	54.2	45.5	42.5	40.7
CV	5.3	5.1	6.1	4.1
Flexural tensile strength (MPa)	5.35	5.92	6.31	6.53
CV	1.5	0.75	0.76	0.81
Splitting tensile strength (MPa)	5.10	5.28	5.45	5.96
CV	0.77	0.71	0.43	0.89
Frost resistance-mass loss after 180 cycles F-T (%)	1.03	1.15	1.43	2.11
CV	0.09	0.09	0.02	0.23
Salt crystallization resistance-loss mass after test (%)	1.00	1.02	1.34	1.79
CV	0.03	0.04	0.07	0.08
Static modulus of elasticity (GPa)	12.93	12.26	11.34	10.91
CV	2.22	1.25	2.31	1.89
Dynamic modulus of elasticity (GPa)	15.47	14.92	13.65	13.12
CV	1.45	1.67	2.32	2.71

CV = Coefficient of variation.
